# Eradication of Biofilm-Mediated Methicillin-Resistant Staphylococcus aureus Infections *In Vitro*: Bacteriophage-Antibiotic Combination

**DOI:** 10.1128/spectrum.00411-22

**Published:** 2022-03-29

**Authors:** Razieh Kebriaei, Katherine L. Lev, Rahi M. Shah, Kyle C. Stamper, Dana J. Holger, Taylor Morrisette, Ashlan J. Kunz Coyne, Susan M. Lehman, Michael J. Rybak

**Affiliations:** a Anti-Infective Research Laboratory, College of Pharmacy and Health Sciences, Wayne State University, Detroit, Michigan, USA; b Center for Biologics Evaluation and Research, US Food and Drug Administration, Silver Spring, Maryland, USA; c School of Medicine, Wayne State University, Detroit, Michigan, USA; Ohio State University

**Keywords:** MRSA, bacteriophages, biofilms

## Abstract

Bacterial biofilms are difficult to eradicate and can complicate many infections by forming on tissues and medical devices. Phage+antibiotic combinations (PAC) may be more active on biofilms than either type of agent alone, but it is difficult to predict which PAC regimens will be reliably effective. To establish a method for screening PAC combinations against Staphylococcus aureus biofilms, we conducted biofilm time-kill analyses (TKA) using various combinations of phage Sb-1 with clinically relevant antibiotics. We determined the activity of PAC against biofilm versus planktonic bacteria and investigated the emergence of resistance during (24 h) exposure to PAC. As expected, fewer treatment regimens were effective against biofilm than planktonic bacteria. In experiments with isogenic strain pairs, we also saw less activity of PACs against DNS-VISA mutants versus their respective parentals. The most effective treatment against both biofilm and planktonic bacteria was the phage+daptomycin+ceftaroline regimen, which met our stringent definition of bactericidal activity (>3 log_10_ CFU/mL reduction). With the VISA-DNS strain 8015 and DNS strain 684, we detected anti-biofilm synergy between Sb-1 and DAP in the phage+daptomycin regimen (>2 log_10_ CFU/mL reduction versus best single agent). We did not observe any bacterial resensitization to antibiotics following treatment, but phage resistance was avoided after exposure to PAC regimens for all tested strains. The release of bacterial membrane vesicles tended to be either unaffected or reduced by the various treatment regimens. Interestingly, phage yields from certain biofilm experiments were greater than from similar planktonic experiments, suggesting that Sb-1 might be more efficiently propagated on biofilm.

**IMPORTANCE** Biofilm-associated multidrug-resistant infections pose significant challenges for antibiotic therapy. The extracellular polymeric matrix of biofilms presents an impediment for antibiotic diffusion, facilitating the emergence of multidrug-resistant populations. Some bacteriophages (phages) can move across the biofilm matrix, degrade it, and support antibiotic penetration. However, little is known about how phages and their hosts interact in the biofilm environment or how different phage+antibiotic combinations (PACs) impact biofilms in comparison to the planktonic state of bacteria, though scattered data suggest that phage+antibiotic synergy occurs more readily under biofilm-like conditions. Our results demonstrated that phage Sb-1 can infect MRSA strains both in biofilm and planktonic states and suggested PAC regimens worthy of further investigation as adjuncts to antibiotics.

## INTRODUCTION

Methicillin-resistant Staphylococcus aureus (MRSA) is the leading species isolated from biofilm-associated infections such as chronic wounds and medical devices. The aggregation of microorganisms inside the protective structure of exopolysaccharides leads to increased bacterial fitness and survival rate due to altered metabolic activity of the biofilm communities, low diffusion of antibiotics inside the biofilm matrix, and blockage of the exposure to immune cells/antibodies (1–4). The narrow pipeline of antibiotics and the development of resistance even to last-resort antibiotics raises the urgent demand for novel antibacterial interventions (5–8). Although biofilms can protect bacteria from harsh environmental conditions and phage predation, some phages encode enzymes, such as depolymerases, that degrade the biofilm extracellular polymeric matrix (EPM) (9, 10). Some of the fundamental differences in the mechanisms of action of phages versus antibiotics also result in phage replication at the site of infection, more specific activity than most antibiotics (potentially reducing dysbiosis), and potentially enhanced tissue and biofilm penetration (10–15). Therefore, phages may have the potential to combat biofilm-associated infections such as persistent chronic infections.

Phage-antibiotic combination (PAC) therapy warrants additional research for several reasons, including that many reported clinical cases of experimental phage treatment include concomitant standard-of-care antibiotics (16). There are indications that PAC may offer therapeutic advantages (i) in the laboratory and some clinical case reports suggest that the emergence of phage-resistance can have trade-off costs leading to antibiotic susceptibility (17–20), and (ii) because additivity and synergy have been observed in some cases with phage-antibiotic combination (PAC) therapy outperforming either agent alone or the expected additive effect of both (12, 18, 21).

Aside from the direct bactericidal effects of PAC, we considered the effects of PAC on other aspects of biofilm biology. S. aureus naturally releases extracellular membrane vesicles (MVs, spherical lipid bilayer structures that contain cytoplasmic components of the cell) into the extracellular environment. Although their exact function and contribution to recalcitrance and persistence are unknown, MVs are known to be key elements and facilitators of biofilm formation (22–24). We previously reported synergy between phage Sb-1 (an S. aureus-specific bacteriophage) and daptomycin (DAP) against a vancomycin-intermediate, daptomycin-resistant S. aureus (VISA-DNS) strain in the planktonic state (25). Here, we expanded that work to focus on biofilms, test additional bacterial strains, and explore the impact of Sb-1 combinations with both DAP and ceftaroline (CPT), a drug that has shown promise against difficult-to-treat S. aureus when added to DAP. Our goals were to (i) determine the activity of PAC against biofilm versus planktonic bacteria, (ii) investigate the emergence of resistance during exposure to PAC in a 24 h exposure period, and (iii) measure the impact of PACs on phage and MV titers in biofilm versus planktonic bacteria.

## RESULTS

### More treatment regimens were effective against the VISA parent strain than an isogenic DNS-VISA mutant strain

**FIG 1:**
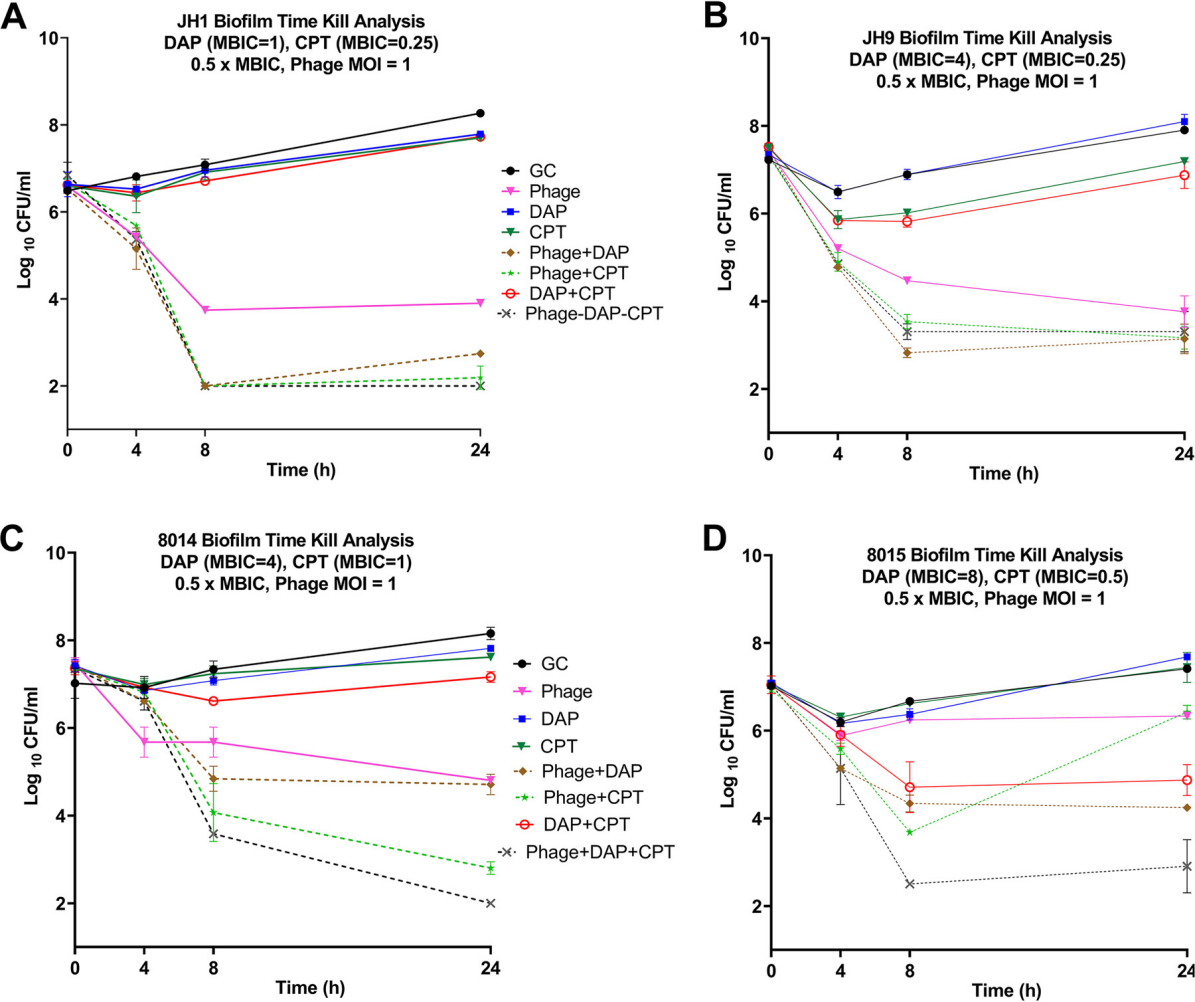
Mean log_10_ CFU/mL versus time values for combinations with DAP and CPT. Solid lines represent single-agent treatments, dashed lines represent PAC treatments. Phage: Sb-1 phage, DAP: daptomycin, CPT: ceftaroline, GC: growth control. Detection limit = 2 log_10_ CFU/mL. SD <0.6 for all graphs.

**(Fig. 1).** To evaluate the impact of phage addition to antibiotic regimens, we investigated two isogenic pairs of patient isolates (JH1 and JH9; 8014 and 8015), in which the parent strains were vancomycin susceptible Staphylococcus aureus (VSSA), and the mutant was VISA (JH-9) or DNS-VISA (8015). Replicate biofilms were established on polystyrene beads before treatment. In these experiments, we used antibiotics at half of the measured minimum biofilm inhibitory concentration (MBIC) for each strain to simulate a situation where treatment failure would occur because the biofilm was not susceptible to the antibiotic regimen being used. As expected, the antibiotic-only treatment regimens had little or no effect on biofilm cell counts.

The combination of phage+DAP+CPT reduced bacterial populations below the detection limit of 2 log_10_ CFU/mL for both parent strains. However, the same combination regimen left slightly higher counts of the mutant strains after 24 h exposure (0.9 and 1.3 log_10_ CFU/mL higher counts for JH9 and 8015, respectively). The overall trend of bactericidal activity was similar in JH1 and JH9 for the same regimens with activity but less bacterial eradication against the mutant. However, in 8014 and 8015, phage+CPT was only effective against the 8014 parents (4.57 log_10_ CFU/mL reduction from initial inoculum) and not against the 8015 mutant (0.5 log_10_ CFU/mL reduction). Phage+DAP and phage alone followed similar patterns in the parent versus mutant strain. This difference in phage activity was not predictable based on plaque assay data because Sb-1 had essentially the same EOP on 8014 and 8015 (Table 1).

**TABLE 1:** List of MIC values in planktonic and biofilm state

Strain	D712		8015		JH1		684		JH9		8014	
Antibiotic	MIC (mg/L)	MBIC (mg/L)	MIC (mg/L)	MBIC (mg/L)	MIC (mg/L)	MBIC (mg/L)	MIC (mg/L)	MBIC (mg/L)	MIC (mg/L)	MBIC (mg/L)	MIC (mg/L)	MBIC (mg/L)
EOP	1		0.95		1.02		1.21		1.02		0.89	
DAP	4	8	4	8	0.25	2	2	4	4	4	0.5	8
VAN	4	8	4	8	1	4	2	8	8	8	2	8
CPT	0.5	4	1	1	0.25	1	0.5	2	0.25	1	1	1

### The synergistic regimens in the planktonic state are not necessarily synergistic in the biofilm state (Fig. 2).

**FIG 2:**
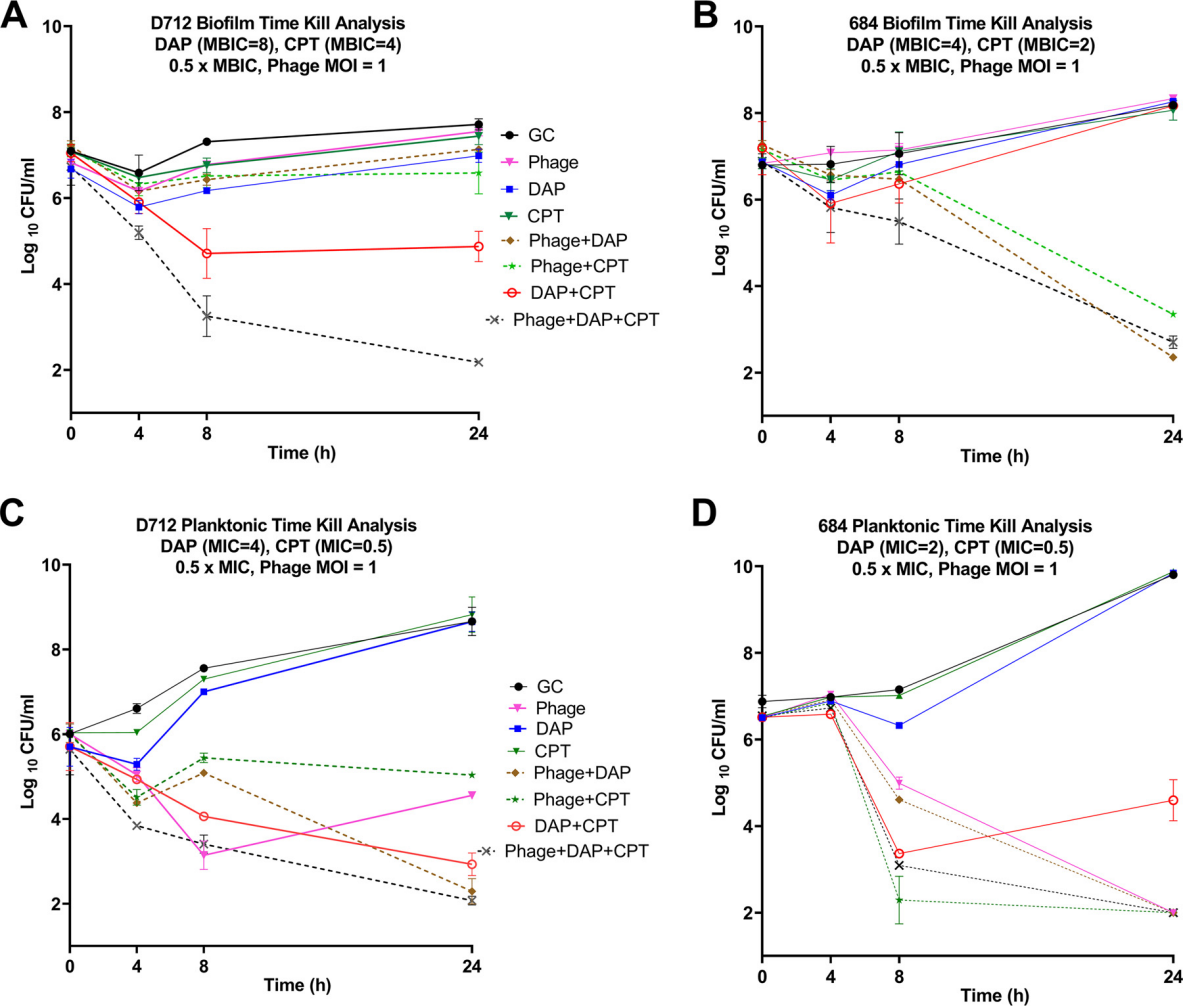
TKA in biofilm state and planktonic state for D712 and 684. Solid lines represent single-agent treatments, dashed lines represent PAC treatments. Phage: Sb-1 phage, DAP: daptomycin, CPT: ceftaroline, GC: growth control. Detection limit = 2 log_10_ CFU/mL. Standard deviation < 0.6 for all graphs.

Sb-1 was highly active on both D712 and 684 in the plaque assay but was better at reducing and suppressing planktonic populations of 684 than D712 in the TKA. Specifically, phage monotherapy reduced 684 counts below the detection limit by 24 h (Fig. 2D), but only caused a 1.5 log_10_ CFU/mL reduction in D712 counts relative to the starting concentration (Fig. 2C) in the planktonic state. Sb-1 alone did not show any impact on biofilm for either organism.

For treatment regimens involving antibiotics, DAP and CPT were used at half of the measured MIC for each strain to simulate a situation where treatment failure would occur (e.g., because the organism *in situ* was less susceptible to the antibiotic than indicated by laboratory testing). Phage+DAP showed synergy (>2 log_10_ CFU/mL greater reduction than the best single agent) against D712 in the planktonic state and for 684 in the biofilm state. This observation is remarkable because both D712 and 684 are DNS and the DAP monotherapy regimen had no activity against these strains in either planktonic or biofilm TKAs. It is also notable that the phage+DAP combination was not synergistic against the DNS-VISA D712 strain in the biofilm state, implying the resilient nature of biofilms even with PAC.

The only phage+antibiotic regimen with bactericidal activity (>3 log_10_ CFU/mL reduction) against D712 biofilm was the combination of phage+DAP+CPT, which reduced bacterial counts almost to the detection limit (Fig. 2A). With strain 684, phage+DAP+CPT was also among the best treatment regimens but was not the only highly effective one (Fig. 2B and D). The combination of phage+CPT reduced planktonic 684 populations below the detection limit slightly faster than the triple combination (Fig. 2D), indicating the potency of this combination against the 684 strain. Interestingly, Fig. 2B shows that nonsusceptibility to DAP is not a determinant of synergy because the phage+DAP combination was effective against 684 biofilms even though neither single agent had an effect.

We did not observe any DAP or CPT MIC reductions in 684 after exposure to phage. No evidence of bacterial resistance to Sb-1 was observed at the end of 24 h in time-kill samples with PAC, whereas phage-alone regimens developed resistance. Additionally, no antibiotic resistance/elevated MICs were observed in any of the regimens at the end of 24 h.

### Titration of phage particles at the end of 24 h treatment was higher in the biofilm state than the planktonic state (Fig. 3A and B).

**FIG 3:**
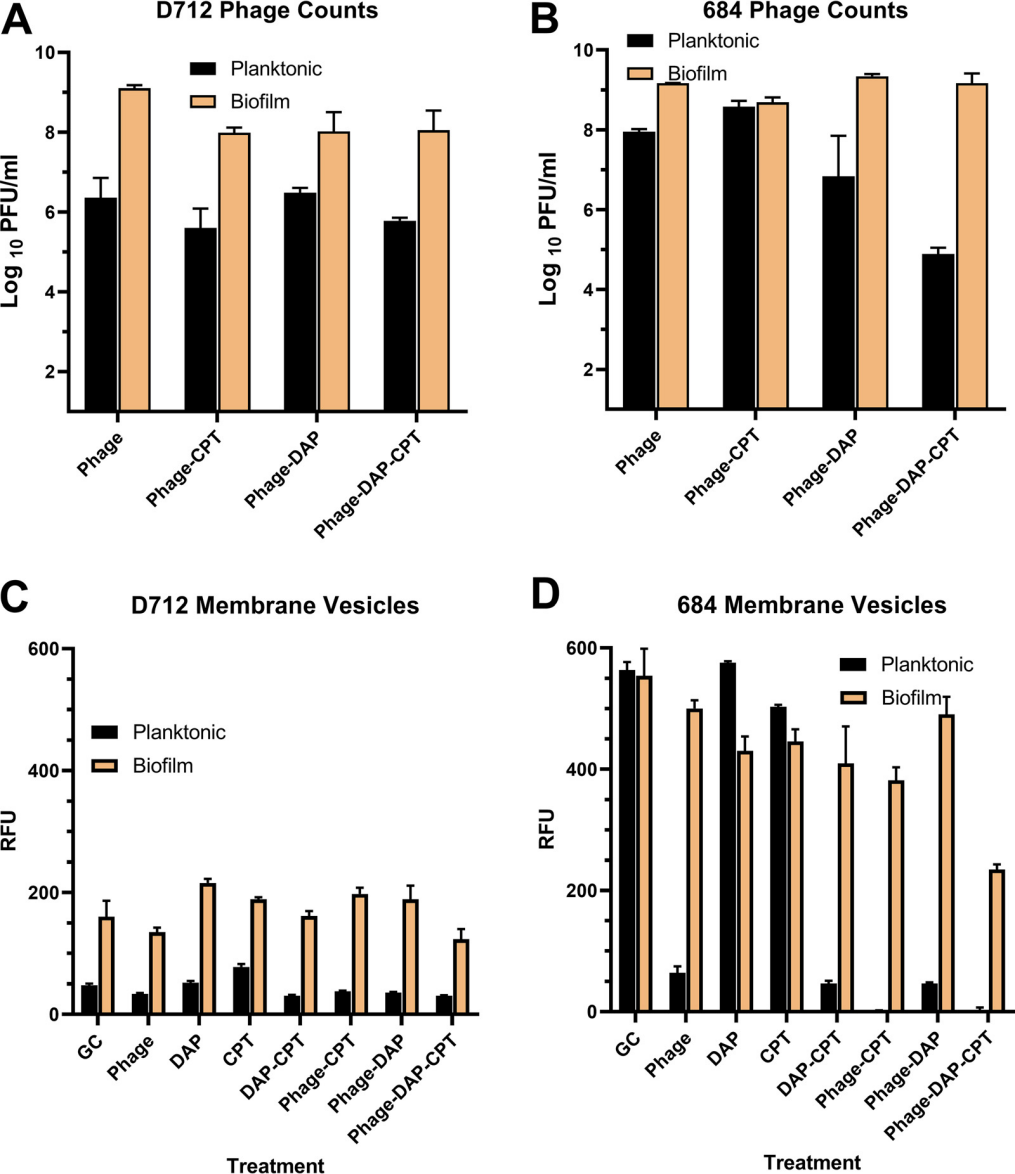
(A and B) Phage titers. (C and D) Relative fluorescence units (RFU) for MV quantifications. Phage: Sb-1 phage, DAP: daptomycin, CPT: ceftaroline, GC: growth control. Standard deviation for all phage counts <1 log_10_ CFU/mL. Standard deviation for all RFU measurements <61 RFU.

While running TKAs, we noticed that phage counts tended to be higher at the end of biofilm experiments than planktonic ones, regardless of treatment regimen or treatment efficacy (e.g., phage alone was only effective against planktonic D712, not biofilm [Fig. 2A and C], but phage concentrations at 24 h were much higher in the biofilm experiment [Fig. 3A]). The difference between phage counts after D712 growth control TKAs was not significant (*P* = 0.08, 0.16 in planktonic and biofilm, respectively). Phage proliferation was not impacted by the addition of antibiotics, except when planktonic 684 was treated with the phage+DAP+CPT regimen (Fig. 3B). Therefore, we hypothesized that there is better Sb-1 proliferation in biofilms than planktonic populations. We tested this hypothesis by designing experiments listed in Table S2. Utilizing beads coated with biofilms we achieved an increase of 3 log_10_ PFU/mL.

### Higher accumulation of membrane vesicles in biofilm state versus planktonic state for most regimens (Fig. 3C and D).

MV formation was intrinsically higher for 684 compared to D712 and both organisms released more MVs in the biofilm state than the planktonic state. In 684 cultures, the apparent reduction in the amount of MVs released in the biofilm state versus planktonic state was not statistically significant (*P* = 0.80). Significant reductions in MV release (*P* = 0.0001) were caused by DAP+CPT as well as all phage-containing treatments in planktonic states of 684. This trend was not observed in D712 planktonic. Although phage had a slight suppressing effect in D712, this difference was not significant (*P* > 0.05).

## DISCUSSION

Several interesting observations emerged in these studies. First, our data demonstrated that the addition of phage to antibiotics prevented phage resistance in the tested strains. Phage resistance was observed in all treatment regimens with Sb-1 alone but was prevented by PACs independent of the S. aureus strain, the extent of virulence, or resistance to antibiotics. Using well-characterized patient isolates, particularly the isogenic pairs, we were able to provide evidence regarding phage resistance prevention by PAC. Kirby (26) also saw that phage+gentamicin combinations prevented phage resistance from emerging in S. aureus, although they only tested a single bacterial strain. While we did not find resensitization (decrease in MIC values after exposure to phage versus before exposure), we identified enhanced bactericidal activity in TKAs when phage Sb-1 was added to the single or double antibiotic combination. Second, our test set of S. aureus strains, while small, was sufficient to show that the triple combination of phage+DAP+CPT was consistently, highly effective across multiple strains and modes of bacterial growth. In contrast, subsets of this combination had variable results with some single or double-agent regimens effective against one strain, but not others, etc. This suggests that there are options for avoiding some of the strain-based variability in PAC efficacy that we have observed (25, 27, 28). Synergy was not dependent on the intrinsic antibiotic susceptibility of the S. aureus strain. This has also been seen for Sb-1 and oxacillin when used in combination against several MRSA strains (29) and may suggest an opportunity to use phages in combination with antibiotics that might not otherwise be considered for use based on classical MIC testing.

Third, we analyzed phage titers both after planktonic and biofilm TKAs to assess the extent of phage proliferation in each setting. Interestingly, we found higher phage titers in biofilm experiments implying sufficient outpace of phage proliferation/infection to bacterial growth in triple combination regimens where the bacterial population is at detection limits. We hypothesized that in regimens with no bactericidal activity phage infections progressed in a relatively constant manner with bacterial growth (i.e., phage alone regimen in biofilm state for both strains) (30). In hopes of discovering the optimized method for phage proliferation, we designed a set of experiments, including biofilm and planktonic bacteria to measure the amount of phage at the end of 24 h, when initial phage and bacteria concentrations were standardized. We discovered that maximum phage titer was harvested from biofilm exposures. While the liquid culture inoculation conditions in this experiment were not necessarily optimized for Sb-1 (using a higher bacterial inoculum than is typical), the magnitude of phage amplification in biofilm was surprising. One possible explanation for this observation may be the compact nature of biofilms which may potentially lead to higher adsorption probability in comparison to submerged planktonic counterparts. Consistent with this explanation, the layer-by-layer three-dimensional structure of biofilms facilitates phage/virion acquirement in a central region leading to increased overall adsorption efficiency (30, 31). This observation is important because biofilms are involved in at least 65% of all bacterial infections and present the biggest challenge for controlling microbial pathogens (32, 33).

Finally, the higher accumulation of MVs during biofilm growth is consistent with previous literature acknowledging MVs as definite components of the biofilm matrix (22). These ambiguous particulates are made of a single cytoplasmic membrane surrounded by a thick cell wall. Having a similar structure to the bacterial cell wall, MVs can potentially bind to/inactivate antibiotics and phages (34, 35). According to previous studies, antibiotics can induce MV release. However, the interaction between phages and MVs requires further investigation (25, 36).

We understand that this study is focused on one phage, but our methods should facilitate broader research on using multiple phages and additional bacterial strains. Furthermore, the emergence of resistance is a time-dependent adaptation and longer exposure times are essential to characterize the impact of PAC on resistance development (37, 38). Moreover, most static *in vitro* models, including our experiments, do not consider the impact of shear stress on biofilm formation which can ultimately affect phage-biofilm interaction. More investigations are needed to address questions regarding the importance of considering treatment order (18), the mechanisms underlying how PAC therapies act, and a systematic evaluation of which combinations are likely to produce desirable or undesirable outcomes.

Despite the limitations, our results provided the groundwork for PAC therapies in the context of multidrug-resistant biofilm-mediated infections. Only the phage+DAP+CPT triple combination was consistently effective across the tested strains, regardless of strain-specific MIC or growth state, and in many cases, this PAC reduced S. aureus populations below our detection limit, even in the biofilm state. Our results support the promising impact of phage+DAP+CPT combinations for further research, including *in vitro* humanized PK/PD models.

## MATERIALS AND METHODS

### Bacterial strains.

Six patient isolates (JH1, JH9, 684, 8014, 8015, D712) were studied in this work. All the strains belong to USA100/ST5 type. JH9 and 8015 are isogenic VISA and DNS-VISA derivatives of JH1 and 8014, respectively (Table S1).

### Antimicrobial agents and media.

DAP was purchased from Merck & Co., Inc. (Whitehouse Station, NJ) and CPT from Allergan Pharmaceuticals (Parsippany, NJ). Colony counts were determined using tryptic soy agar (TSA). Mueller-Hinton broth II (MHB) (Difco, Detroit, MI, USA) with 25 mg/L calcium and 12.5 mg/L magnesium was used for susceptibility testing. For all experiments with DAP, an additional 25 mg/L of calcium was added to the broth due to the dependency of DAP on calcium for antimicrobial activity. Phage propagation and testing were done using heart infusion broth (HIB; BD Bacto, San Jose, CA, USA) with 1.5% agar (Oxoid, Lenexa, KS, USA) for underlays and 0.7% agar for overlays. Tryptic soy broth (TSB; Difco, Detroit, MI) supplemented with 1% glucose (GSTSB) was used for biofilm TKAs.

### Susceptibility testing.

Minimum biofilm inhibitory concentration (MBIC) values were determined in duplicate using the pin-lid method (formerly referred to as Calgary Biofilm Device) (39, 40). Briefly, biofilms were grown on plastic pins for 18 to 24 h followed by antibiotic susceptibility testing via the broth microdilution method (BMD) following Clinical and Laboratory Standards Institute (CLSI) guidelines (41). Combination MBIC values for DAP in the presence of CPT were determined by supplementing the broth with concentrations of CPT at half MIC.

### Time-kill experiments.

Time-kill analyses (TKA) were performed using DAP and CPT at 0.5× MIC or MBIC (depending on whether it is planktonic or biofilm TKA) values to simulate subinhibitory concentrations. Phage dosing was optimized to a multiplicity of infection (MOI_input_) ratio of 1, which represents the ratio of input phage particles to the target organism. The experiment was performed in duplicate in 24-well tissue-culture-treated plates with 2 mL of broth and 4 sterile polystyrene beads in each well. Plates were incubated in a shaker incubator at 37°C for 24 h to allow for biofilm growth on the beads in 1% glucose supplemented tryptic soy broth (GSTSB). After 24 h of incubation, GSTSB was aspirated and replaced with MHB. Antibiotic and phage were then added after the first (0 h) sampling. One bead was taken for sampling and processing at 0, 4, 8, and 24 h to create a growth curve.

Collected samples were washed with 1 mL of sterile saline to remove planktonic bacteria and processed for 6 min, alternating 1 min each of sonication and vortex to disrupt the attached biofilm. The bead was removed sterilely and samples containing phage were then centrifuged and filtered to isolate phage and bacteria from each other for counting. The collected bacterial samples were serially diluted appropriately and plated using automatic spiral platers (easySpiral, Interscience for Microbiology, Saint Nom la Breteche, France) with a detection limit of 10^2^ CFU/mL. Plates were incubated for 18 to 24 h of growth at 37°C and bacterial colonies were counted using a laser colony counter (Scan 1200, Interscience for Microbiology, Saint Nom la Breteche, France). Collected phage samples were counted using the phage quantification protocol described below. Antibiotic and phage carry-over was addressed by centrifugation and/or serial dilutions of the samples.

Bactericidal activity was defined as >3 log_10_ CFU/mL reduction from baseline. The synergy between two agents was defined as a >2 log_10_ CFU/mL reduction at 24 h compared to the most active agent alone. Regarding triple combinations, simply the log_10_ CFU/mL reduction was reported.

### Bacteriophages, source, and propagation.

Sb-1 was isolated on S. aureus D712 from a bacteriophage solution purchased from Georgia Eliava Institute (Tbilisi, Georgia). Sb-1 is a myophage that belongs to the *Herelleviridae* family according to ICTV Master Species List number 36. Phage genomic DNA was isolated by treating the filtered lysates with DNase and RNase to remove bacterial nucleic acids, followed by proteinase K treatment and organic extraction. A PCR-free genomic library was prepared and sequenced (Illumina MiSeq, PE150 reads). Trimmed reads (adapters removed, minimum base quality = 30) were assembled using Unicycler. The single-copy genome of our isolate is at least 137,661 bp. Read mapping conducted in Geneious Prime (Biomatters Ltd.) revealed 8076 bp direct terminal repeats (DTRs) as a region of approximately doubled coverage bounded by sharp cliffs. Therefore, a packaged genome of at least 145,737 bp is predicted. This is larger than the published genome of Sb-1 (NC_023009 [42]), almost entirely due to differences in the DTRs. The DTRs in Sb-1 were predicted to be only 3959 bp, missing several genes that are present in the DTRs of our isolate and closely related phages. Our isolate also differs in the copy number of the iteron repeat. Additional work is needed to accurately resolve this region in our Sb-1 isolate, but our read data mapped to NC_0023009 showed substantially increased coverage in this region, indicating that more copies are present in our isolate.

Sb-1 bacteriophage was propagated to obtain high titer stocks to use in resistance testing and time-kill analyses. To begin, an underlay layer of 1.5% HIB agar was poured into square Petri plates. A 6 mL overlay of 0.7% HIB agar was immediately combined with 100 μL of an overnight host S. aureus bacterial culture containing approximately 10^9^ CFU/mL and poured atop the underlay layer. The overlay was briefly allowed to set, and following this, 750 μL of purified bacteriophage was spread over top and incubated in a 37°C incubator overnight. The overlay agar was scraped into 3 mL of phosphate-buffered saline (PBS) + 10 mM magnesium sulfate and centrifuged at 1000 rpm for 25 min at 4°C. The supernatant was filtered and stored covered at 2 to 8°C for experimental use.

### Phage sensitivity assays.

Bacterial sensitivity to the Sb-1 phage was checked using the small-drop agar overlay method (43) where 10-fold serial dilutions of phage were spotted onto 0.7% HIB overlay plates containing an overnight culture of the target bacteria. Plates contained 6 mL of overlay agar which was briefly mixed with 100 μL of overnight culture and left to dry for 10 min before spotting 5 μL of purified phage onto the bacterial lawn. Sensitive strains were further evaluated via plaque assay to determine the efficiency of plating (EOP) as a ratio of PFU on the test strain to PFU on the reference strain (ATCC 43300) (44).

### MV quantification.

MV samples were taken at 24 h from corresponding wells in the TKA. Each sample tube was centrifuged (12000 rpm, 5 min) and the supernatant was filtered (pore size 0.2 μm) and frozen –20°C until ready to be tested. Samples from growth control and media control were also tested as positive and negative controls, respectively. This process was tested and repeated to purify the MVs from bacterial cells and phage particles present in the sample.

Purified MV suspensions were quantified spectrofluorometrically as described previously (Bolt method) with modifications (36, 45). MV was detected on a SpectraMax M5 instrument using a membrane-specific dye (FM1-43, Life Technologies Corporation, 5 μg/mL, ex/em 485/560 nm).

### Resistance tests.

Resistance screening was performed using the double-drop method (43, 44). Briefly bacteriophage insensitive mutants (BIMs) were generated by mixing 100 μL of the bacterial culture (sample) (at concentration PFU:CFU of about 10:1) with 100 μL of concentrated phage and left to sit at room temperature for 10 min. Then 3 mL of 0.7% overlay will be added to the plate and incubated for 48h. The number of colonies in this plate will demonstrate an apparent frequency of resistance. Each colony will be cultured in a 3 mL snap cap tube for 6 to 8 h and using the double drop method 5 μL of the resulting culture will be spotted on top of 10 μL high titer phage with PFU/CFU of about 10:1. After 24 h, any emergent colony was counted as resistant. Antibiotic resistance tests were performed as described previously against DAP and CPT (46, 47).

### Statistical analysis.

Statistical analysis was performed using one-way analysis of variance with Tukey’s multiple-comparison test (with *P* < 0.05 considered significant). All the graphs and statistical analysis were prepared in Prism 8, Version 8.4.3, GraphPad.
