# Supplementary material for: Eradication of Biofilm-Mediated Methicillin-Resistant Staphylococcus aureus Infections In Vitro: Bacteriophage-Antibiotic Combination
Source: Microbiol Spectr. 2022 Mar 29;10(2):e00411-22. doi: 10.1128/spectrum.00411-22 (PMC9045164; doi:10.1128/spectrum.00411-22)
Supplement: SUPPLEMENTAL FILE 1 — Supplemental material. Download SPECTRUM00411-22_Supp_1_seq12.pdf, PDF file, 0.04 MB [file spectrum00411-22_supp_1_seq12.pdf]

Supplementary Material

Table.1S: List of pair strains with their EOP to Sb-1 and their MIC values.

			EOP	MIC		
**Isolate**	**Phenotype**	**Strain Type**	**Sb-1**	**VAN²**	**DAP**	**CPT**
**D712**	DNS-VISA	USA100/ST5	1	4	4	0.5
**D592**	hVISA	USA100/ST5	0.869855	2	0.5	2
**8015**	DNS-VISA	USA100/ST5	0.946242	4	4	1
**8014**	MRSA	USA100/ST5	0.893186	2	0.5	1
**684**	DNS	USA100/ST5	1.206012	2	4,2	0.5
**675**	MRSA	USA100/ST5	1.174767	0.5	<0.13	0.5
**JH9**	VISA	USA100/ST5	1.015945	8	1	0.5
**JH1**	VSSA	USA100/ST5	1.028677	1	0.25	0.25
**306**	DNS-VISA	USA300/ST8	1.039276	4,2	4	0.5
**305**	MRSA	USA300/ST8	1.015945	1	0.25	0.5
**R6913**	VISA	USA100/ST5	1.02263	8	4	1
**R6911**	hVISA	USA100/ST5	1.039276	2	2	1

Table 2S. Experimental design for various propagation method

Experiment details (CFU and PFU are at time of phage addition)	MOI/phage quantity	Average PFU/mL
Planktonic time kill (10^6 CFU/mL+10^5 PFU/mL)[1]	0.1	$4*10^6$
Liquid (phage and bacteria, 10^7 CFU/mL+10^6 PFU/mL)[2]	0.1	$1.4*10^8$
Liquid culture (phage and bacteria, 10^7 CFU/mL+10^7 PFU/mL)[3]	1	$3.4*10^6$
GSTSB (after biofilms are formed on the beads, 10^7 of bacteria in biofilm CFU/mL+10^6 PFU/mL)[4]	0.1	$1.2*10^7$
GSTSB (after biofilms are formed on the beads, 10^7 of bacteria in biofilm CFU/mL+10^7 PFU/mL)[5]	1	$9*10^5$
Biofilm-MHB<br>After biofilms are formed on the beads (24 h culture) and GTSB is replaced with MHB (10^7 of bacteria in biofilm CFU/mL+10^6 PFU/mL)[6].	0.1	$5.2*10^{10}$

[1]TKA plate well: 1.98 mL MHB + 20 µL (MOI 0.1), incubate for 24 h in shaker incubator, aspirate 1 mL from well for phage propagation.
[2]Overnight liquid culture: 1 colony D712 into 3 mL HIB, incubate for 16-18 hours in shaker incubator, add 100 µL of overnight culture and 20 µL phage (MOI 0.1) to 9.88 mL MHB broth and incubate for 24 hours in shaker incubator, aspirate 1 mL for phage propagation.
[3]Overnight liquid culture: 1 colony D712 into 3 mL HIB, incubate for 16-18 hours in shaker incubator, add 100 µL of overnight culture and 20 µL phage (MOI 1) to 9.88 mL MHB broth and incubate, for 24 hours in shaker incubator, aspirate 1 mL for phage.
[4]TKA plate well: 1.98 mL TSB, incubate for 24 h, add 20 µL phage (MOI 0.1), incubate for 24 h in shaker incubator, remove 1 mL from well for phage propagation.
[5]TKA plate well:
1.80 mL TSB, incubate for 24 h, add 200 µL phage (MOI 1), incubate for 24 h in shaker incubator, remove 1 mL from well for phage propagation.

[6]TKA plate well: 2 mL TSB, incubate for 24 h, aspirate TSB and replace with 1.98 MHB, add 20 µL phage (MOI 0.1), incubate x 24 hours in shaker incubator, aspirate 1 mL from well for phage propagation.
[7]Round HIB plate: add 100 µL D712 overnight culture to 3 mL HIB overlay and pour over HIB plate, allow to dry for 10 minutes. Pour 400 µL phage (10^9 PFU/mL) over plate, incubate for 24 h, scrape lawn with T loop and place in 3 mL PBS buffer. Centrifuge for 2 min, remove and filter supernatant for phage propagation.
Abbreviations: GSTSB, glucose-supplemented tryptic soy broth; MHB, Mueller-Hinton broth
